# *Mycobacterium bovis* genomics reveals transmission of infection between cattle and deer in Ireland

**DOI:** 10.1099/mgen.0.000388

**Published:** 2020-06-18

**Authors:** Joseph Crispell, Sophie Cassidy, Kevin Kenny, Guy McGrath, Susan Warde, Henrietta Cameron, Gianluigi Rossi, Teresa MacWhite, Piran C. L. White, Samantha Lycett, Rowland R. Kao, John Moriarty, Stephen V. Gordon

**Affiliations:** ^1^​ School of Veterinary Medicine, University College Dublin, Dublin, Ireland; ^2^​ Data Science Campus, Office for National Statistics, Newport, UK; ^3^​ Central Veterinary Research Laboratory, Backweston, County Kildare, Ireland; ^4^​ UCD Centre for Veterinary Epidemiology and Risk Analysis (CVERA), School of Veterinary Medicine, University College Dublin, Dublin, Ireland; ^5^​ Royal (Dick) School of Veterinary Studies, University of Edinburgh, Edinburgh, UK; ^6^​ Roslin Institute, University of Edinburgh, Edinburgh, UK; ^7^​ Department of Agriculture, Food and the Marine, Backweston, County Kildare, Ireland; ^8^​ Department of Environment and Geography, University of York, Wentworth Way, York YO10 5NG, UK; ^9^​ UCD Conway Institute of Biomolecular and Biomedical Research, University College Dublin, Dublin, Ireland

**Keywords:** bovine tuberculosis, *Mycobacterium bovis*, deer, badger, Wicklow, phylogenetics

## Abstract

Control of bovine tuberculosis (bTB), caused by *Mycobacterium bovis *, in the Republic of Ireland costs €84 million each year. Badgers are recognized as being a wildlife source for *M. bovis* infection of cattle. Deer are thought to act as spillover hosts for infection; however, population density is recognized as an important driver in shifting their epidemiological role, and deer populations across the country have been increasing in density and range. County Wicklow represents one specific area in the Republic of Ireland with a high density of deer that has had consistently high bTB prevalence for over a decade, despite control operations in both cattle and badgers. Our research used whole-genome sequencing of *M. bovis* sourced from infected cattle, deer and badgers in County Wicklow to evaluate whether the epidemiological role of deer could have shifted from spillover host to source. Our analyses reveal that cattle and deer share highly similar *M. bovis* strains, suggesting that transmission between these species is occurring in the area. In addition, the high level of diversity observed in the sampled deer population suggests deer may be acting as a source of infection for local cattle populations. These findings have important implications for the control and ultimate eradication of bTB in Ireland.

## Data Summary

All whole-genome sequence data used for our analyses have been uploaded to the National Center for Biotechnology Information Sequence Read Archive (NCBI-SRA) under BioProject number PRJNA589836: www.ncbi.nlm.nih.gov/bioproject/PRJNA589836. Due to the sensitivity of the associated metadata, only the sampling date and species are provided with these sequences. All the code generated for this manuscript is freely available on GitHub: scripts to process the whole-genome sequencing data https://github.com/JosephCrispell/GeneralTools/tree/master/ProcessingPipeline; and scripts used to analyse the processed genomic data – https://github.com/JosephCrispell/GeneralTools/tree/master/RepublicOfIreland/Wicklow.

**Impact Statement**

In the Republic of Ireland, bovine tuberculosis (bTB), caused by *Mycobacterium bovis *, threatens the sustainability of cattle production, with bTB control costing the government and industry €84 million per year. Whilst badgers are recognized as being a source of infection for cattle, similar evidence on the role of deer in Ireland is lacking – despite the known susceptibility of deer to *M. bovis *. Whole-genome sequencing of *M. bovis* has previously been used to elucidate the role of different host species in multi-host pathogen transmission systems. Here, we use whole-genome sequencing of *M. bovis* sourced from infected cattle, badgers and deer to investigate the role of deer in the spread and persistence of *M. bovis* infection in a bTB hotspot in County Wicklow, Ireland. Our analyses suggest that *M. bovis* is transmitted between cattle and deer populations, and that deer may be acting as an important source of infection in the area. As such, *M. bovis* genome sequencing can shed new light *on M. bovis* transmission and provide quantitative data to support bTB policy formulation.

## Introduction

Bovine tuberculosis (bTB), caused by *Mycobacterium bovis *, affects cattle populations around the world [1–4]. In many countries with endemic bTB, wildlife play a role in the spread and persistence of *M. bovis* infection in cattle, hence, complicating bTB control [3, 5–8].

In the Republic of Ireland, control of bTB currently costs farmers, the exchequer and the European Union €84 million per year [9]. Populations of the European badger (*Meles meles*) can maintain *M. bovis* and act as a source of infection for cattle [10, 11]. As a result, badger populations across the country are managed as part of the national bTB control programme [12]. While deer are susceptible to infection, their role in *M. bovis* spread and persistence is uncertain due to insufficient data, and deer are not managed nationally under the bTB control programme [13–15].

The epidemiological role of deer in bTB, i.e. whether they are spillover hosts or a source of infection, is known to be linked to population density [7, 16–21]. Infection outcomes in deer range from a relatively common presentation of minimal pathology with infected deer living for many years, to a rarer chronic generalized infection involving multiple organ systems and a high fatality rate [7, 22–25]. Across Europe, deer species such as red deer and fallow deer are known to act as sources of infection for cattle in localized areas of high density, or as part of a multi-host wildlife reservoir [17, 18, 26, 27]. In Ireland, bTB outbreaks in Irish farmed deer have also been documented [28]. One bTB ‘hot-spot’ in Ireland is County Wicklow, where high densities of deer have been implicated in the local spread and persistence of *M. bovis* infection in cattle [29]. Furthermore, the range and density of wild-deer populations in Ireland is increasing [30, 31]. These increases highlight the need to quantify the role of deer in bTB epidemiology [32].

Whole-genome sequencing of *M. bovis* has been used to track transmission within and between cattle and wildlife populations [21, 33–37]. These studies have demonstrated that genomics adds unprecedented resolution, in comparison to previous molecular-typing technologies, in many cases distinguishing infection between individual animals. With Ireland seeking eradication of bTB by 2030 [9], the additional resolution of genomics could provide critical insights about transmission within and between cattle and wildlife populations, and hence serve to support and refine bTB control policy.

A key question in resolving the current bTB hotspot in County Wicklow is to establish whether wild deer are involved in the spread and persistence of *M. bovis* in the local cattle population. Herein, we describe the application of whole-genome sequencing of *M. bovis* sampled from infected cattle, badgers and deer taken from a 100 km^2^ area in County Wicklow to directly address this question.

## Methods

### Sample selection

**Fig. 1.**
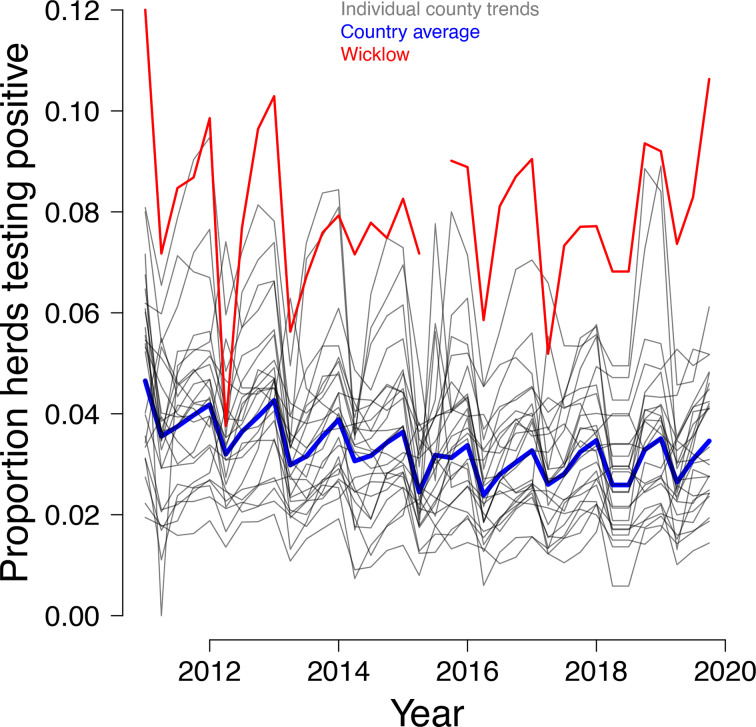
Proportion of herds in each county of the Republic of Ireland testing positive for bovine tuberculosis from 2011 to 2020. Source: ‘Bovine Tuberculosis by Regional Veterinary Offices, Year and Statistic (2010-498 2019)’.

In the last decade, County Wicklow has frequently had the highest herd-level prevalence of bTB in the Republic of Ireland, as shown by Fig. 1 [38, 39]. The Irish Department of Agriculture, Food and the Marine (DAFM) conducted a research study in County Wicklow in 2014 and 2015 that aimed to establish the prevalence of *M. bovis* infection in the local deer population (J Moriarty and others, unpublished data). During this study, culling operations were conducted in the deer and badger populations, while cattle herds in the area underwent statutory bTB testing. *M. bovis* culture was performed using MGITs (mycobacteria growth indicator tubes) from post-mortem samples of the culled badgers and deer, and test-positive cattle. Positive cultures were archived at the DAFM Central Veterinary Research Laboratory (Backweston, Ireland).

**Fig. 2.**
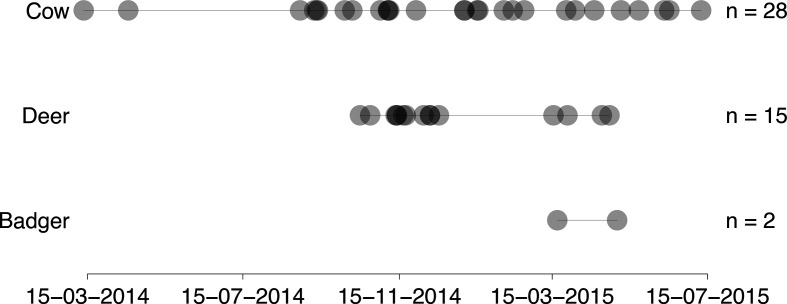
Sampling dates for the *M. bovis* samples available from the Wicklow area. Shading is darker where circles overlap.

**Fig. 3.**
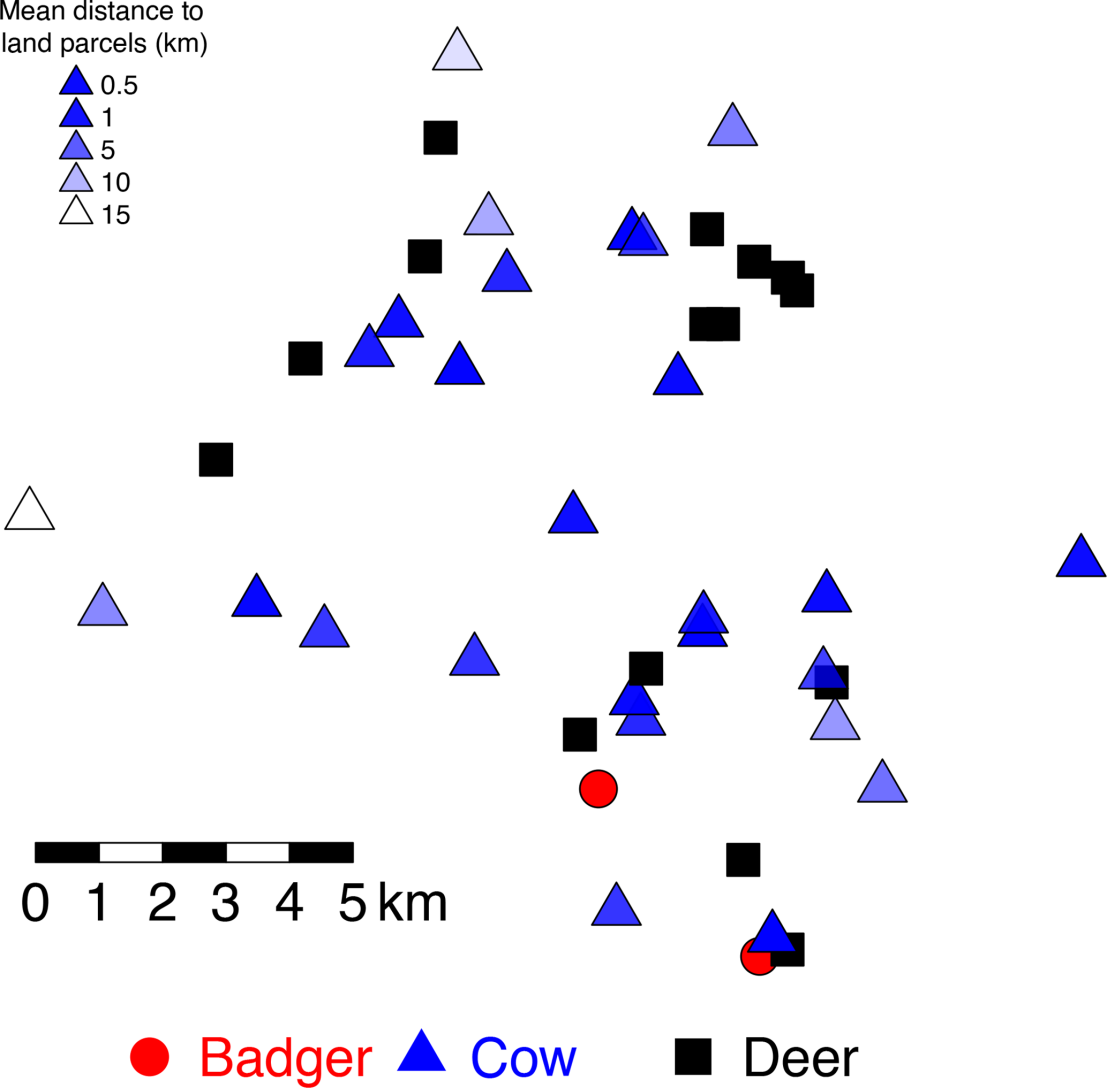
Sampling locations for the *M. bovis* isolates from the Wicklow area. Each point represents the capture location for deer, the sett for badgers and the approximate herd locations for cattle [the latter to protect the identity of farm owners in compliance with GDPR (General Data Protection Regulation)]. The transparency of shading for cattle locations illustrates our certainty about where the sampled cow resided: the more transparent the triangle the more distant the herd’s land parcels were from the approximate location.

Frozen isolates from cattle, deer and badgers within the selected time frame were located in the DAFM Central Veterinary Research Laboratory archives and re-cultured for sequencing. While the majority of deer and cattle isolates were resuscitated, only a minority of badger isolates could be recovered. The sequenced isolates represent all deer and badger isolates that could be recovered from the biobank, which were originally collected from 133 deer (23 infected on culture) and 68 badgers (17 infected on culture). The 28 cattle isolates that were sequenced were sampled from a total of 274 cattle isolates from this region, from which 174 isolates were available, with a single isolate from each herd selected for whole-genome sequencing; this isolate was from a home-bred animal or an animal that had been in the herd for several years. Hence, in total, 45 *M *. *bovis* isolates were successfully re-cultured from 28 cattle, 15 deer (14 sika and 1 fallow) and 2 badgers, sampled from 2014 to 2015 (Fig. 2). All the samples were sourced from animals present within an area of approximately 100 km^2^, equating to approximately 5 % of the total area (2027 km^2^) of County Wicklow (Fig. 3). Wildlife locations were provided as coordinates of the location where the animal was shot or trapped (DAFM). Cattle locations were derived from land-parcel data associated with each sampled herd available from the DAFM Land Parcel Identification System (LPIS). Cattle testing information was available through the DAFM Animal Health Computer System (AHCS).

### Whole-genome sequencing data – generation and processing

DNA was extracted from the cultured *M. bovis* isolates using an AMPure XP magnetic bead based extraction protocol [40] and sequenced at the UCD Conway Institute Genomics Core (Dublin, Ireland) using an Illumina NextSeq system, producing 2×150 bp paired-end reads. The raw sequencing data was assessed using fastqc (v0.11.2; RRID:SCR_014583) [41]. The sequencing reads were trimmed, and adapters were removed where present using Cutadapt (v1.18; RRID:SCR_011841) [42]. Trimmed reads were aligned against the *M. bovis* reference genome (AF2122/97) [43] using the mem tool from bwa (Burrows–Wheeler aligner) (v0.7.17; RRID:SCR_010910) [44]. Any annotated repeat regions, or those encoding proline-glutamic acid (PE) and proline-proline-glutamic acid (PPE) proteins, were excluded [45]. Excluding the single nucleotide variants (SNVs) within the PE and PPE regions was found to have no influence on the phylogenetic relationship reported in the current research (Supplementary Figs 1 and 2, available with the online version of this article). For the aligned sequence data, SNVs were recorded if they had mapping quality ≥30, high-quality base depth ≥4 on the forward and ≥4 on the reverse reads, read depth ≥30 reads and allele support ≥0.95. If a site failed these criteria and the allele called was observed in each isolate’s sequence data, it was accepted if it had a total high-quality base depth ≥4 and allele support ≥0.95. Any SNVs within 10 bp of one another were removed to avoid regions of the genome that were prone to sequencing errors or under high selection. All the genomes were *in silico* spoligotyped using the *SpoTyping* tool (v2.0; RRID:SCR_018466) [46] .

### Phylogeny reconstruction

A maximum-likelihood phylogeny was reconstructed with RAxML (v8.2.11; RRID:SCR_006086) [47] using an alignment based on the concatenated SNVs from each sequenced isolate with a generalized time-reversible (GTR) substitution model [48]. The phylogeny was visualized in the statistical programming environment R (v3.6.1) [49] using the ape package (v5.0; RRID:SCR_017343) [50].

### Clustering

The extent of species-level clustering in the genetic distances between the *M. bovis* genomes was investigated. Genetic distances were calculated by counting the number of differences between each pair of concatenated SNV sequences. These genetic distances were then divided into within- and between-species categories and compared.

## Results

### Whole-genome sequencing

High-quality sequencing data was generated for all 45 *M *. *bovis* isolates [on average, each genome had 99 % coverage (lower 2.5 %, 0.82; upper 97.5 %, 0.99) of its genome with a read depth ≥20 reads]. Spoligotypes could be reconstructed from the whole-genome sequence data and all isolates were type SB00054.

### Phylogeny in space

**Fig. 4.**
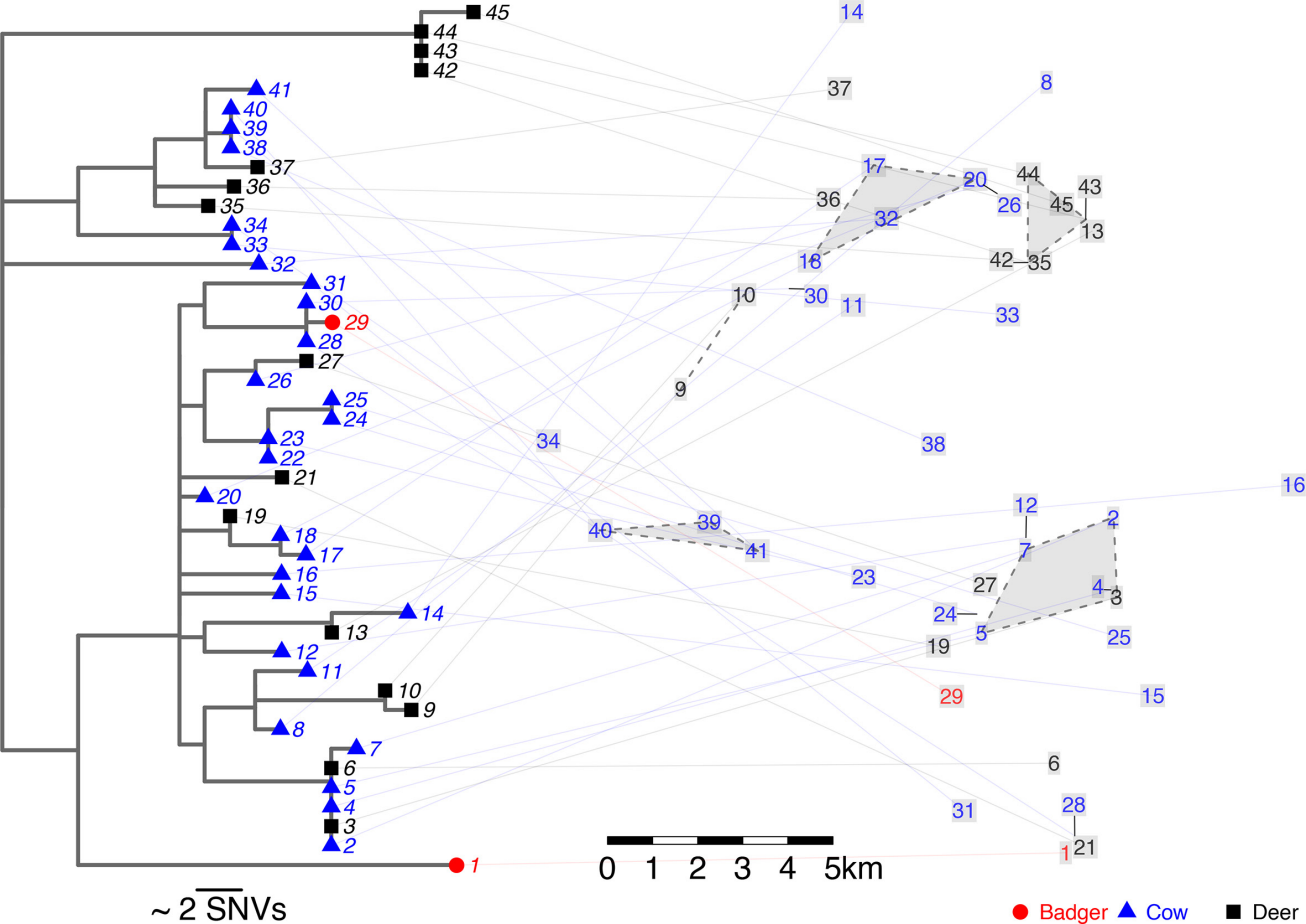
A maximum-likelihood phylogeny built with RAxML (v8.2.11) [47] and rooted using AF2122/97 (* M. bovis* reference genome) [43]. Each of the tips is linked via a line to its sampling location. The sampling locations are plotted as the indices of the tip in the phylogeny. Some sampling locations were slightly repositioned to avoid overlapping labels using the basicPlotteR R package (https://github.com/JosephCrispell/basicPlotteR). Grey polygons highlight clusters of genomes with three or less differences and with approximate sampling locations within 2.5 km.

All the *M. bovis* genomes sourced from infected cattle, badgers and deer in the Wicklow area were within 35 SNVs of one another (median distance=14 SNVs; lower 2.5 %, 1; upper 97.5 %, 30) (Fig. 4). There were multiple instances of *M. bovis* genomes sourced from cattle and wildlife being less than three SNVs apart, a distance that, in the human field, is indicative of recent transmission of *Mycobacterium tuberculosis* [51]. The deer-derived *M. bovis* genomes had the highest genomic diversity, with representatives found across the phylogeny as well as in a distinct single-species clade (labels 42–45 in Fig. 4).

The approximate sampling locations for the cattle, badgers and deer were all within 17 km of one another (median distance, 6.7 km; lower 2.5 %, 1.1; and upper 97.5 %, 12.9). The polygons in Fig. 4 highlight where animals that were infected with highly similar strains (≤3 SNVs) of *M. bovis* were found in close proximity (≤2.5 km) to one another. Only four small clusters were identified, suggesting that, in general, animals sharing similar *M. bovis* were not sampled close to one another. One of the clusters identified contained both cattle and deer (labels 2–5 and 7 in Fig. 4).

### Patterns of clustering

**Fig. 5.**
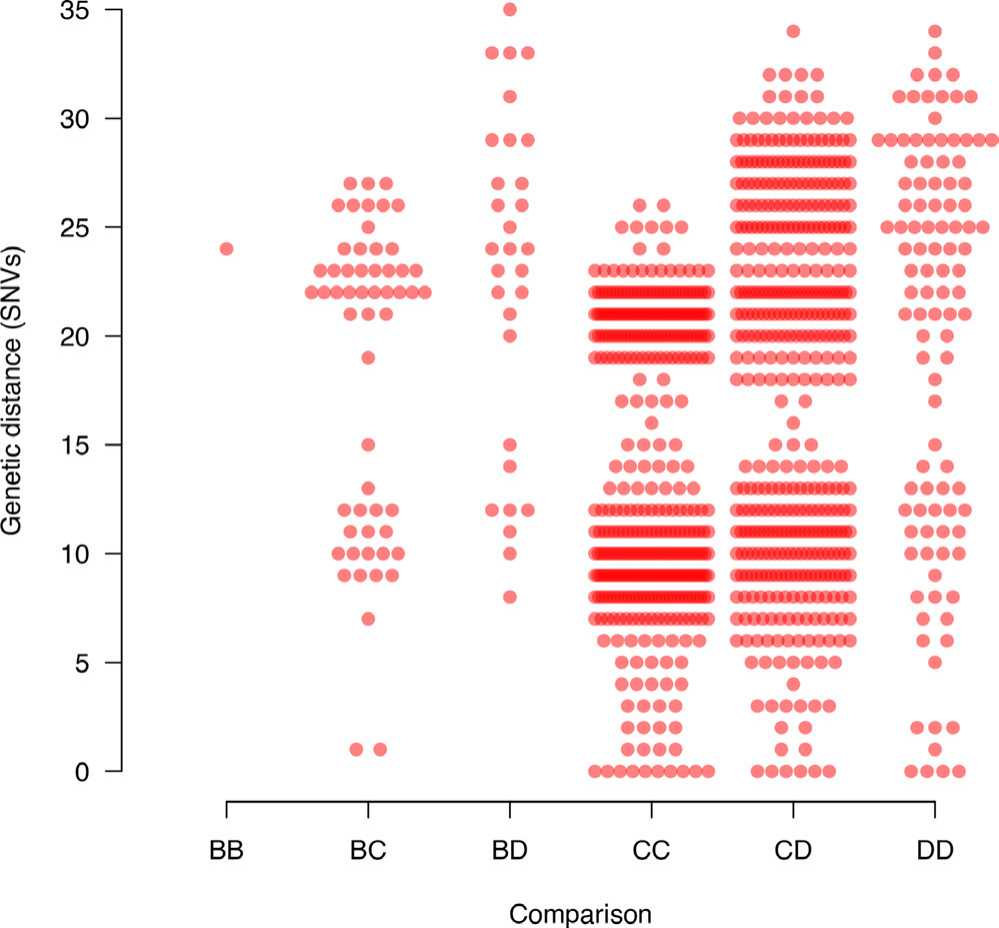
Comparing the genetic distances within and between species. The genetic distance distribution for the *M. bovis* genomes was subdivided into distances associated with badger–badger (BB), badger–cattle (BC), badger–deer (BD), cattle–cattle (CC), cattle–deer (CD) and deer–deer (DD) comparisons. The raw data were overlaid using the spreadPoints() function in the basicPlotteR R package (https://github.com/JosephCrispell/basicPlotteR).

There was no evidence of species-specific clustering in the genetic distance distribution, since there was considerable overlap between all the within- and between-species genetic distance distributions (Fig. 5). The multiple instances of cattle- and wildlife-derived *M. bovis* genomes being highly similar (<3 SNVs) are shown in the badger–cattle and cattle–deer subsets of the genetic distance distribution.

## Discussion

Our research used *M. bovis* whole-genome sequence data to address whether deer in County Wicklow have an important epidemiological role in bTB in cattle. Analysis of the *M. bovis* genomes sourced from cattle, badgers and deer found that all species shared highly similar strains. Our data are limited, and the following interpretation is, therefore, framed within these constraints.

The high similarity of the *M. bovis* genomes sourced from cattle, badgers and deer suggests that in the sampled area all three host species are involved in the spread and persistence of *M. bovis* (Fig. 3). While badgers are a recognized source of *M. bovis* for cattle, and the presence of a badger-derived *M. bovis* genome only one SNV from two cattle-derived strains supports this (Fig. 5), the availability of only two *M. bovis* isolates from badgers limits our ability to further examine their role. In contrast, the larger number of samples from deer presents evidence suggesting recent transmission between cattle and deer, with 5 of the 15 *M *. *bovis* genomes sourced from deer being within three or less SNVs of those sourced from cattle (Figs 4 and 5). Importantly, such similarity could result from a common source, such as badgers. Defining the role of deer in the bTB system in Wicklow will require further research for which our study provides the baseline.

Despite having 28 genomes sourced from cattle, there was more diversity between the deer-derived *M. bovis* genomes (Fig. 5). Within-species diversity is commonly used to evaluate the epidemiological role of species, with high diversity suggesting the species is acting as a source of infection [52]. While concluding that deer are acting as a source population in County Wicklow would rely upon representative sampling of each host species, which was not possible here, the diversity of *M. bovis* in deer suggests they could be playing an important role in the spread and persistence of infection in the area.

If deer are playing a role in the cattle and wildlife *M. bovis* transmission systems, local persistence of *M. bovis* in wildlife could be prolonged and infection spread further. Sika and fallow deer can live up to twice as long as badgers (12–16 years versus 5–8 years in badgers) and range over considerably larger distances (mean home-range size of 0.5–10 km^2^ versus typically less than 500 m for badgers) [53–57]. These large ranging distances could explain the spatial clustering shown in Fig. 4 **,** which suggests that the *M. bovis* strains present in the area are not spatially constrained. The aggregation of cattle, deer and badgers into herds and social groups means infection can persist locally. However, deer are recognized as important spatial vectors for *M. bovis* spread [7, 13, 20, 58–60] and their movements could be spreading *M. bovis* within the sampling area, hence, reducing patterns of spatial localization.

Combating pathogen transmission in a multi-host system requires knowledge of each host species’ role [61]. Our research shows how *M. bovis* genome sequencing has the potential to provide new and detailed insights into local transmission dynamics in the bTB system. In Ireland, badgers are known to be a maintenance host of *M. bovis* infection. Our current research suggests that in County Wicklow deer could also be acting as a source of infection for cattle, potentially as part of a multi-host wildlife reservoir similar to those that exist with wild boar [26]. Therefore, our research highlights the need for surveillance to extend to deer populations in areas of high density across Ireland and provides a compelling case for the integration of genomics into routine bTB surveillance.

## Data Bibliography

1. Crispell J, Cassidy S, Kenny K, McGrath G, Warde S, Cameron H, Rossi G, MacWhite T, White PCL, Lycett S, Kao RR, Moriarty J, Gordon SV. Whole-genome sequence data, NCBI-SRA BioProject number PRJNA589836 (www.ncbi.nlm.nih.gov/sra/PRJNA589836) (2019).

2. Crispell J. GitHub, scripts to process the whole-genome sequencing data – https://github.com/JosephCrispell/GeneralTools/tree/master/ProcessingPipeline (2017).

3. Crispell J. GitHub, scripts to analyse the processed genomic data – https://github.com/JosephCrispell/GeneralTools/tree/master/RepublicOfIreland/Wicklow (2020).

## Supplementary Data

Supplementary material 1
