# Supplementary material for: Mycobacterium bovis genomics reveals transmission of infection between cattle and deer in Ireland
Source: Microb Genom. 2020 Jun 18;6(8):mgen000388. doi: 10.1099/mgen.0.000388 (PMC7641417; doi:10.1099/mgen.0.000388)
Supplement: Supplementary material 1 [file mgen-6-388-s001.pdf]

Supplementary Information: *Mycobacterium bovis* genomics reveals transmission of infection between cattle and deer in Ireland

1.1 Author names

Joseph Crispell*[1,9] (https://orcid.org/0000-0002-0364-7112),
Sophie Cassidy[1],
Kevin Kenny[2],
Guy McGrath[3](https://orcid.org/0000-0002-8545-396X),
Susan Warde[2],
Henrietta Cameron[2],
Gianluigi Rossi[5] (https://orcid.org/0000-0002-6540-8467),
Teresa MacWhite[4] (https://orcid.org/0000-0002-3164-0073),
Piran C. L. White[6](https://orcid.org/0000-0002-7496-5775),
Samantha Lycett[7] (https://orcid.org/0000-0003-3159-596X),
Rowland R. Kao[5] (https://orcid.org/0000-0003-0919-6401),
John Moriarty[2] &
Stephen V. Gordon[1,8] (https://orcid.org/0000-0002-4833-5542)
*Author for correspondence

1.2 Affiliation

1. School of Veterinary Medicine, University College Dublin
2. Central Veterinary Research Laboratory, Backweston, Co. Kildare, Ireland
3. UCD Centre for Veterinary Epidemiology and Risk Analysis (CVERA), School of Veterinary Medicine, University College Dublin
4. Department of Agriculture, Food and the Marine, Backweston, Co. Kildare, Ireland
5. The Roslin Institute and Royal (Dick) School of Veterinary Studies, University of Edinburgh
6. Department of Environment and Geography, University of York, Wentworth Way, York YO10 5NG
7. The Roslin Institute, University of Edinburgh
8. UCD Conway Institute of Biomolecular and Biomedical Research, University College Dublin
9. Data Science Campus, Office for National Statistics, United Kingdom

1.3 Corresponding author

crispelljoseph@gmail.com

1.4 Keyword

Bovine tuberculosis, *Mycobacterium bovis*, deer, badger, Wicklow, phylogenetics

1.5 Repositories:

Whole genome sequence data uploaded to the National Centre for Biotechnology Information Short Read Archive (NCBI-SRA): https://www.ncbi.nlm.nih.gov/bioproject/PRJNA589836.

1. Comparing sequences with/without PE/PPE regions

During the processing of the whole genome sequence data for the *M. bovis* isolates used in the current research (described in main manuscript: **Section 6.2**), the regions encoding Proline-Glutamic acid (PE) and Proline-Proline-Glutamic acid (PPE) proteins were excluded. These regions comprise approximately 10% of the *M. bovis* genome and are known to be highly variable (Sampson 2011). The PE and PPE proteins these regions encode are thought to play a role in how the bacteria interacts with its host, and the variability of these regions provides a mechanism by which the bacteria can evolve and evade the host immune response (Nair 2014). As a result of the high variability of these regions, for phylogenetic analyses that assume a uniform distribution of variability across the genome, Single Nucleotide Variants (SNVs) falling within these regions are generally excluded (Phelan et al. 2016).

To investigate the influence of excluding SNVs falling within the PE/PPE regions on the phylogenetic relationships described in the main manuscript, we repeated the construction of the phylogeny using sequences that did not exclude any variation present in these regions. **Figure 1** demonstrates that the inclusion of these regions made no influence on the phylogenetic relationships described in the main manuscript. Although the positions of three of the tips have changed, the phylogenetic relationships remain consistent. **Figure 2** corroborates this result showing that there is a perfect correlation between the genetic distances calculated with and without the PE/PPE regions (confirmed with a mantel test).

2. References

Nair, Shiny. 2014. “Immunomodulatory Role of Mycobacterial PE/PPE Family of Proteins.” *Proceedings of the Indian National Science Academy*. https://doi.org/10.16943/ptinsa/2014/v80i5/47973.

Phelan, Jody E., Francesc Coll, Indra Bergval, Richard M. Anthony, Rob Warren, Samantha L. Sampson, Nicolaas C. Gey van Pittius, et al. 2016. “Recombination in Pe/ppe Genes Contributes to Genetic Variation in Mycobacterium Tuberculosis Lineages.” *BMC Genomics* 17 (February): 151.

Sampson, Samantha L. 2011. “Mycobacterial PE/PPE Proteins at the Host-Pathogen Interface.” *Clinical & Developmental Immunology* 2011 (January): 497203.

3. Figures and tables

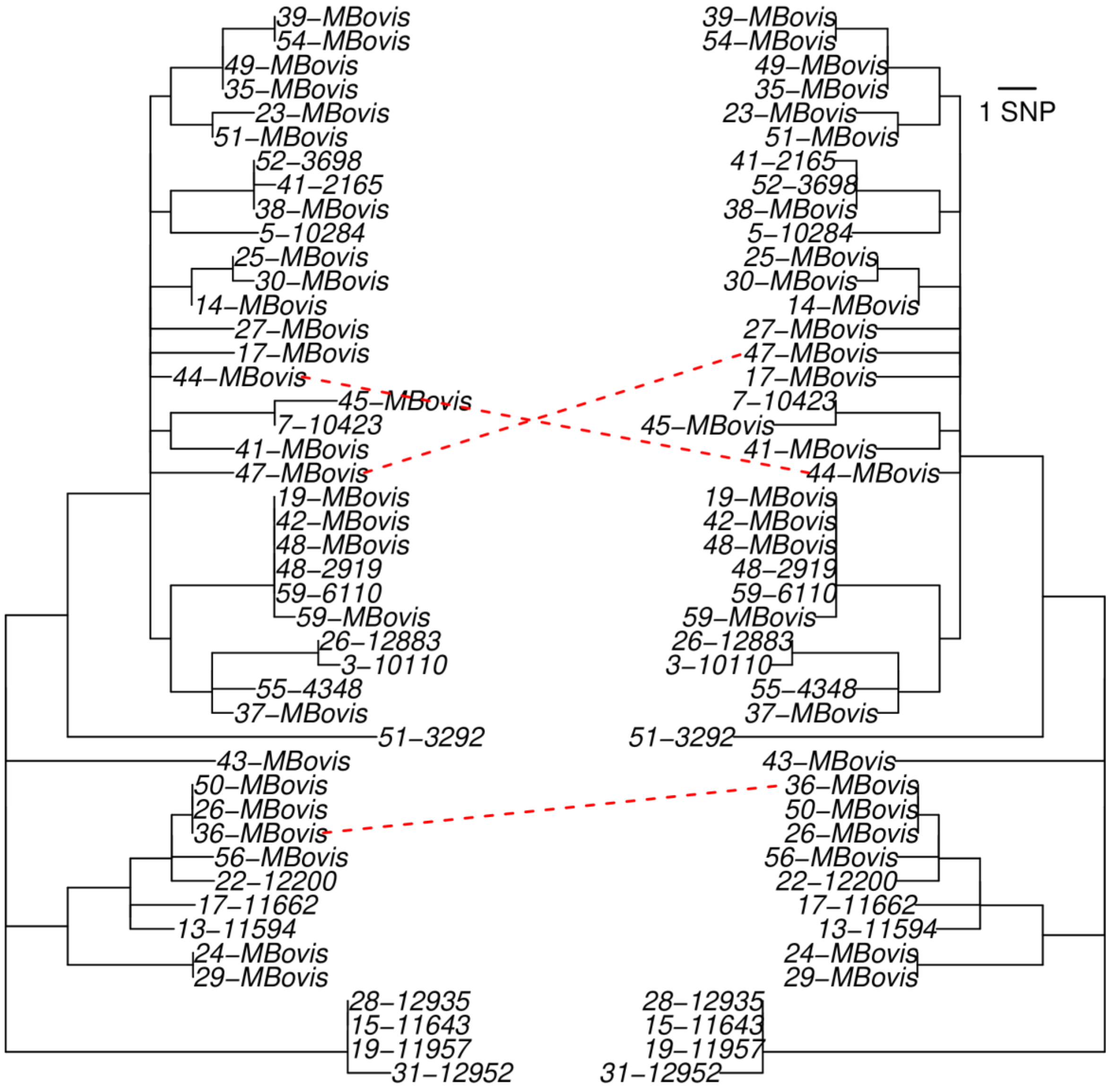

Figure 1: A tangle plot comparing a phylogeny built with (LEFT) and without (RIGHT) SNVs in the PE/PPE regions.

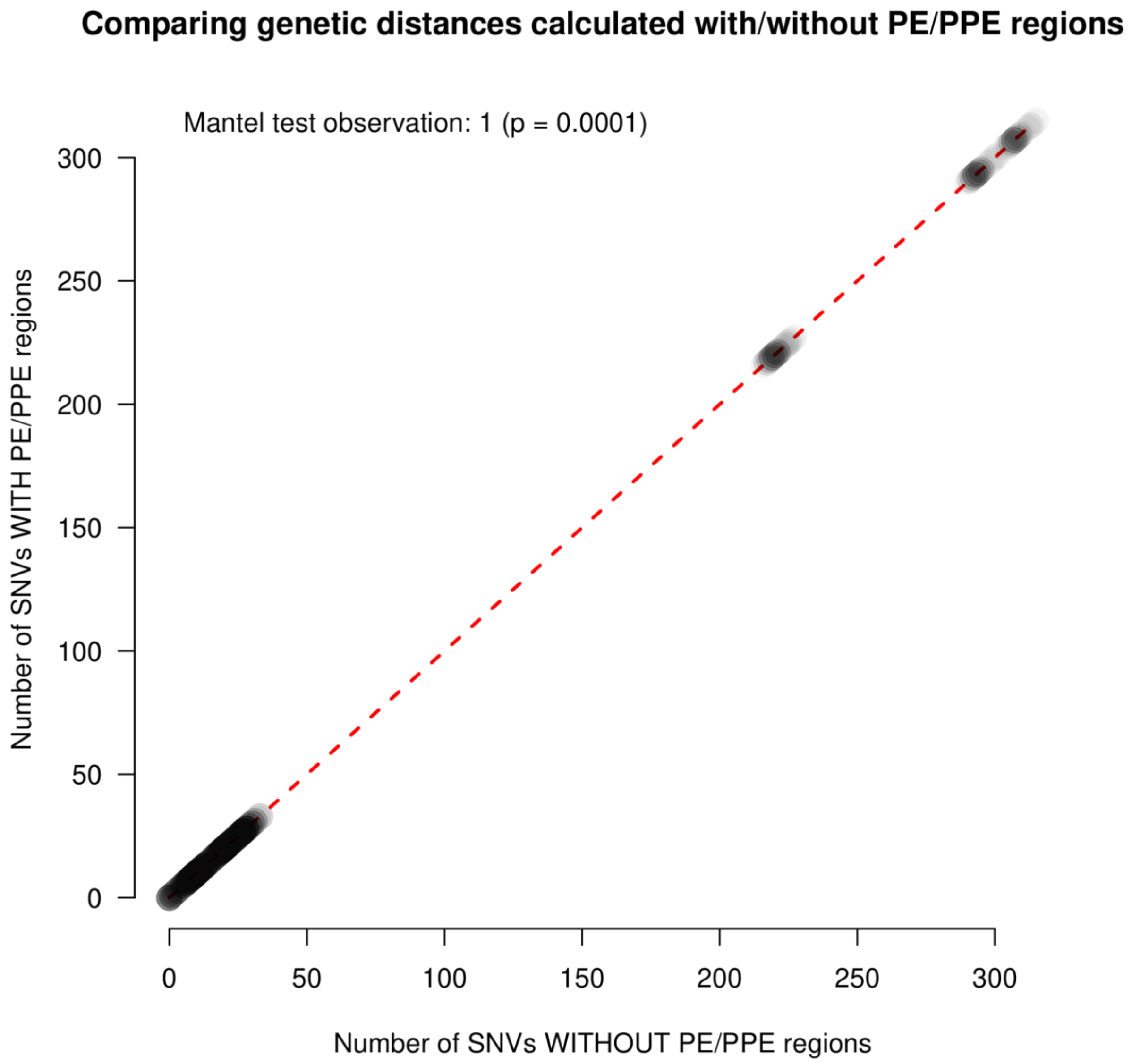

Figure 2: Correlation between the genetic distances calculated with (Y axis) and without (X axis) the PE/PPE regions. The correlation between these distances was quantified using a mantel test.
